# Wireless Biological Electronic Sensors

**DOI:** 10.3390/s17102289

**Published:** 2017-10-09

**Authors:** Yue Cui

**Affiliations:** College of Engineering, Peking University, Beijing 100871, China; ycui@pku.edu.cn; Tel.: +86-10-8252-4860

**Keywords:** wireless, biological electronic, sensor, electrode, signal, detection

## Abstract

The development of wireless biological electronic sensors could open up significant advances for both fundamental studies and practical applications in a variety of areas, including medical diagnosis, environmental monitoring, and defense applications. One of the major challenges in the development of wireless bioelectronic sensors is the successful integration of biosensing units and wireless signal transducers. In recent years, there are a few types of wireless communication systems that have been integrated with biosensing systems to construct wireless bioelectronic sensors. To successfully construct wireless biological electronic sensors, there are several interesting questions: What types of biosensing transducers can be used in wireless bioelectronic sensors? What types of wireless systems can be integrated with biosensing transducers to construct wireless bioelectronic sensors? How are the electrical sensing signals generated and transmitted? This review will highlight the early attempts to address these questions in the development of wireless biological electronic sensors.

## 1. Introduction

Biological electronic sensors have attracted increasing interests due to the advantages of being miniaturized, portable, specific, and sensitive, and they can be used for a variety of applications, such as medical diagnosis/prognosis [1,2], drug detection [3,4], defense/warfare [5,6], and food contamination [7,8]. Here, a biological electronic sensor that the review focuses on is an integrated electronic device based on the immobilization of biological molecules on a sensing electrode for the detection of a target analyte [9,10]. Though there are several studies for developing wireless electronic devices without the immobilization of biological molecules for biology application [11,12,13,14,15,16], this review does not focus on these sensing systems. The target analyte interacts with immobilized bioreceptors on the surfaces of the sensing electrode that further induces a change in an electrical signal, such as conductance [17], current [18,19], potential [20,21], frequency [22,23], phase [24,25], amplitude, impedance [26,27], and/or capacitance [28]. The signal response can be monitored and correlated to the concentration of the target analyte through a calibration curve. Therefore, when there is a signal response from an unknown concentration of an analyte, the concentration can be determined from the calibration curve.

Most sensors usually require a connection between the sensors and the external instruments with cables [29,30,31,32]. In the presence of an analyte, the interaction between the target analyte and the bioreceptor on the sensing electrode induces a change of an electrical signal, and through the connection cable, the signal can be transmitted to the external instrument. These sensors provide excellent sensitivities and rapid responses. However, the connection cables limit the applications. Thus, it is highly desirable to develop wireless biological electronic sensors. The wireless sensors can provide the advantages of being wearable, real-time, continuous, in vivo sensing, or long-distance sensing.

This review focuses on recent progress in wireless biological electronic sensors. To construct a wireless biological electronic sensor, a bioreceptor-immobilized sensing transducer is integrated with a wireless antenna. This paper will first review the biosensing transducers that have been used in the construction of wireless biological electronic sensors, and then explore different methods for the construction of wireless bioelectronic sensors. The wireless biological electronic sensing systems are classified into the following categories: wireless radio-frequency identification (RFID)-based biosensors, wireless acoustic wave-based biosensors, wireless magnetoelastic biosensors, wireless self-powered biosensors, and wireless potentiostat-based biosensors.

## 2. Biological Electronic Sensing Transducers

To develop a wireless biological electronic sensor, a sensing transducer is immobilized with bioreceptors to construct a biosensing transducer. The biosensing transducer is further integrated with a wireless communication element to transmit the sensing signals to an external receiving device.

Several types of sensing transducers have been used for the construction of biological electronic sensors. These include electrochemical electrodes [2,33,34], transistors [1,35,36], resistors [37], capacitors [38], surface acoustic wave (SAW)-based electrodes [39,40,41], magnetic acoustic plates [42], and magnetoelastic ribbons [43,44]. The bioreceptors mainly include catalytic-based bioreceptors and binding/hybridization based bioreceptors. The catalytic-based bioreceptors can be enzymes [33,45] or cells [46,47]. The binding/hybridization-based bioreceptors can be antibodies [48,49], DNA [50,51], peptides [52,53], or phages/viruses [44,54,55].

Among different types of electronic sensing transducers, electrochemical electrodes are widely used as the sensing transducers for the construction of a variety of biosensors [2,33,34,56]. An amperometric sensor, which consists of a working electrode, a reference electrode, and a counter electrode, is a typical widely-used electrochemical sensor. These sensors can be based on a three-electrode configuration or a two-electrode configuration. For the two-electrode configuration, one electrode is the working electrode, and the other electrode functions as both a reference and counter electrode. A potentiostat maintains the potential between the working electrode and the reference electrode, by adjusting the current at the counter electrode. The working electrode is immobilized with a specific bioreceptor. In the presence of a target analyte, the biological reaction on the working electrode generates a change in either a direct-current (DC) signal or alternating-current (AC) signal. Electrochemical electrodes can be used as the electrodes in biofuel cells, too, and the analyte-related biological reactions on the electrode can generate electrical current.

Capacitors, especially interdigital electrode-based capacitors, are used as the sensing elements in biosensors [38]. Capacitors are based on AC signals, and can be used in binding/hybridization-based biosensors. In the presence of an analyte, a sensing signal response can be generated by changing the electrical properties of the capacitor via the biological reaction on the capacitor.

Several types of transistors have been studied as the transducers of biological electronic sensors [1,35,36]. The gate for the transistor can be a bottom gate or an electrolyte gate. Different parts in the transistor can function as the sensing elements, including the channel, the gate electrode, or the dielectric layer. For most studies, the bioreceptors are generally immobilized on the channels to develop biological electronic sensors.

Resistor-based biosensors have some similarities with field-effect transistors. When there is no gate potential, a bottom-gate field-effect transistor can function as a resistor. The bioreceptors are immobilized on the resistors, and both catalytic-based biological reactions or binding/hybridization-based biological reactions can be used for the resistor-based biosensors [37]. The biological reactions on the resistors can result in changes of the resistances to generate signal responses.

A surface acoustic wave (SAW) is generated from a high-frequency electromagnetic wave on the surface of a piezoelectric material. There are two main ways of wave transmission. The first is a delay line, where the wave propagates to another inter-digital transducer where it is converted to electrical signal. The second is a reflective delay line, where the wave propagates towards reflectors and back to the inter-digital transducers. There are one-port and two-port resonators. A one-port resonator consists of one electrically-connected inter-digital transducer and all other elements are reflective. Similarly, a two-port resonator has two electrically-connected ports, one is the input port, and the other is the output port. SAWs can be used in passive sensors and biosensors [39,40,41].

Magnetic acoustic sensors use aluminized silica discs, quartz, or other materials that can generate transverse acoustic waves by electromagnetic fields as the sensing plates [42]. The acoustic waves resonate between the upper and the lower surfaces. The bioreceptors are immobilized on the sensing plate, and in the presence of an analyte, the property of the acoustic wave can be changed to generate a signal.

Magnetoelastic material, such as an amorphous ferromagnetic alloy, shows a characteristic resonance frequency as a function of the dimension, shape, and mass of the material [57]. When an alternating magnetic field is applied with a frequency that matches this characteristic resonance frequency, a maximum shape change of the magnetoelastic material can occur, and this can be monitored with a surrounding magnetic pick-up coil connected to an external frequency-monitoring device. Magnetoelastic materials have been studied for the construction of wireless bioelectronic sensors [43,44].

These electronic devices are further functionalized with bioreceptors. Biological molecules can provide high specificities, though some of them have low stabilities. The biological reactions can generally be an enzyme-based catalytic reaction, cell-based catalytic reaction, antibody-antigen-based binding, peptide-based binding, deoxyribonucleic acid (DNA)-based hybridization, cell-based binding, and phage/virus-based binding. For a variety of small molecules, enzymatic reactions can be used to convert the analytes into other molecules that further result in electron transfers on sensing electrodes [33,45]. For large molecules, antibody-based binding reactions are often used for the detection, and the analytes can change the resistances or the impedances of the sensors [48,49]. To detect DNA or ribonucleic acid (RNA), a sensor with immobilized complementary DNA, RNA, or peptide nucleic acid (PNA) is generally used for detection through hybridization [50,51], and during hybridization, the DNA or RNA analyte with negative charges can be brought onto the sensing electrodes to result in signal responses. For many molecules ranging from small to large sizes, specific peptides can be selected for binding to the analytes [52,53]. Bacteria or virus, themselves, can be used as the bioreceptors, such as bacteria that can be used to detect biochemical oxygen demand (BOD) [46,47] that can generate signals on the sensing electrodes through metabolic reactions, and viruses can capture specific analytes onto viruses [58].

## 3. Wireless RFID-Based Biological Electronic Sensors

To wirelessly transmit the sensing signals from a sensing transducer to a receiver, which is in range, passive RFIDs have been integrated with bioelectronic sensing units. Inductors are widely used to integrate with capacitors to construct inductor-capacitor (LC) or inductor-capacitor-resistor (LCR) circuits as the sensing devices [22,59,60], and the construction of the entire device structure is simple and straightforward. Dipole antennas have also been studied for wireless transmission of sensing signals [57,61].

**Figure 1:**
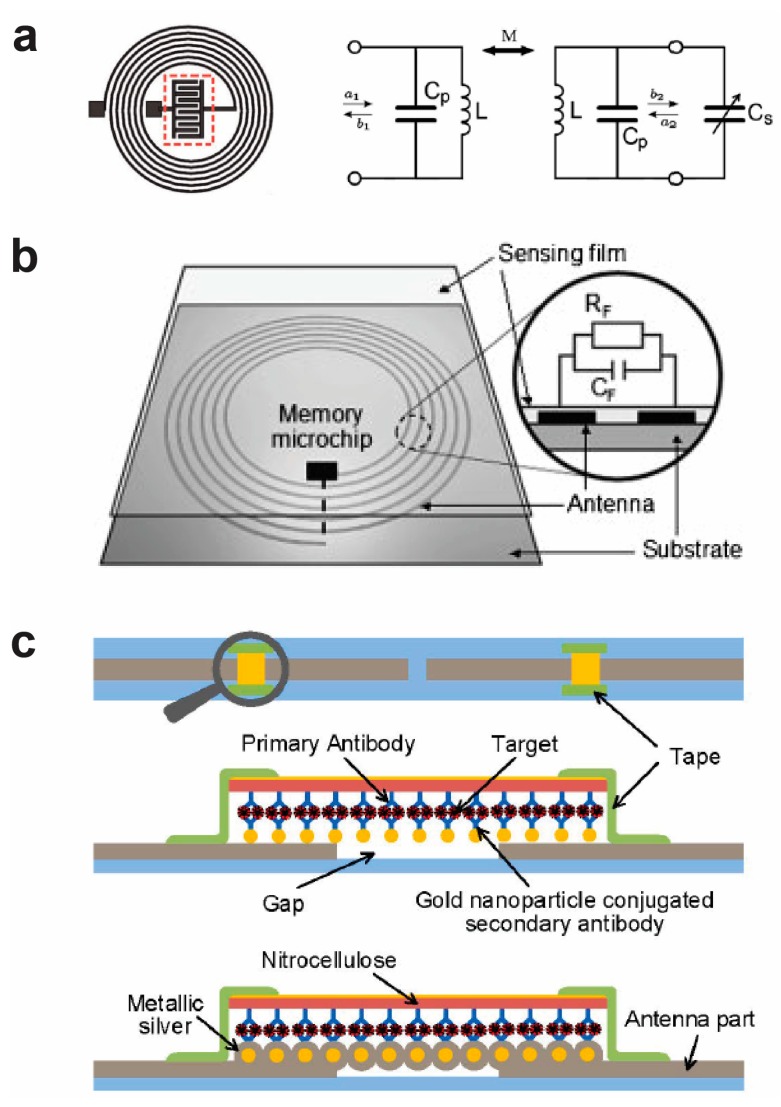
Wireless inductor-based wireless bioelectronic sensors. (**a**) Inductor-capacitor elements (left) and equivalent circuit model of the inductor-capacitor sensor (right). Reprinted with permission from [60]. Copyright (2013) Wiley. (**b**) LC tag connected to a memory microchip. Reprinted with permission from [62]. Copyright (2009) Wiley. (**c**) The silver enhancement process for a dipole antenna biosensor. Reprinted with permission from [57]. Copyright (2015) IEEE.

Figure 1a shows a LCR-based sensor that uses an inductor coupling to a capacitor [60], and its equivalent circuit model. An external coil is connected to an external instrument, and the instrument generates an oscillating current in the coil that further generates a magnetic field. By bringing the external coil near to the inductor in the LCR sensor, there is a mutual induction between the two coils when the operating frequency is near its resonance frequency, and the energy can be transferred to the sensor coil. In the presence of analyte, the binding of the analyte to the capacitor in the LCR device changes the electrical properties of the LCR device, such as permittivity, impedance, resonance frequency, and S11.

In addition to studying the properties of the LCR device under different frequencies, the RFID device can be directly connected to a memory microchip and operated at a fixed frequency, 13.56 MHz, which is a widely used for wireless communication [62]. The detection is based on depositing a sensing film onto the resonant antenna, and the analyte changes the resistance or capacitance of the sensing film which, in turn, changes the impedance, as shown in Figure 1b.

In some situations, the binding of a target analyte with a concentration is not sufficient to generate large enough changes in the measuring properties, such as S-parameters. Therefore, in order to detect the target analyte, a silver enhancement with conjugated gold nanoparticles is used for the detection. Figure 1c shows the principle of the silver enhancement process for the dipole antenna biosensor [57]. During the antigen-antibody binding process for the detection of antigen, gold nanoparticles are used as labels. Further, the labels induce the silver enhancement process, and the silver ions are reduced on the gold nanoparticles and the size of the shell grows. When the size of the shell is large enough, the insulation properties of the gap become electrically conductive.

RFID-based sensors have simple configurations that can be easily fabricated or printed on a variety of substrates. The transmission of the wireless signal generally occurs only over a short distance. Therefore, the receiver must be near to the sensor. This is a limitation of the RFID-based bioelectronic sensor. Until now, though there are several types of RFID-based chemical sensors that have been developed [59,60,63], there are very few studies on LCR-based wireless bioelectronic sensors. The manufacturing of RFID biosensor is compatible with mass production, and it has been widely used for a variety of other applications. It is anticipated that RFID-based devices with a variety of configurations will be developed in the future.

## 4. Wireless Acoustic Wave-Based Biological Electronic Sensors

Converting an electronic signal to an acoustic signal is another method to construct a wireless biological electronic sensor. This section mainly focuses on two types of acoustic-based sensors. The first one is based on surface acoustic wave (SAW). The second one is magnetic acoustic resonance biosensor.

**Figure 2:**
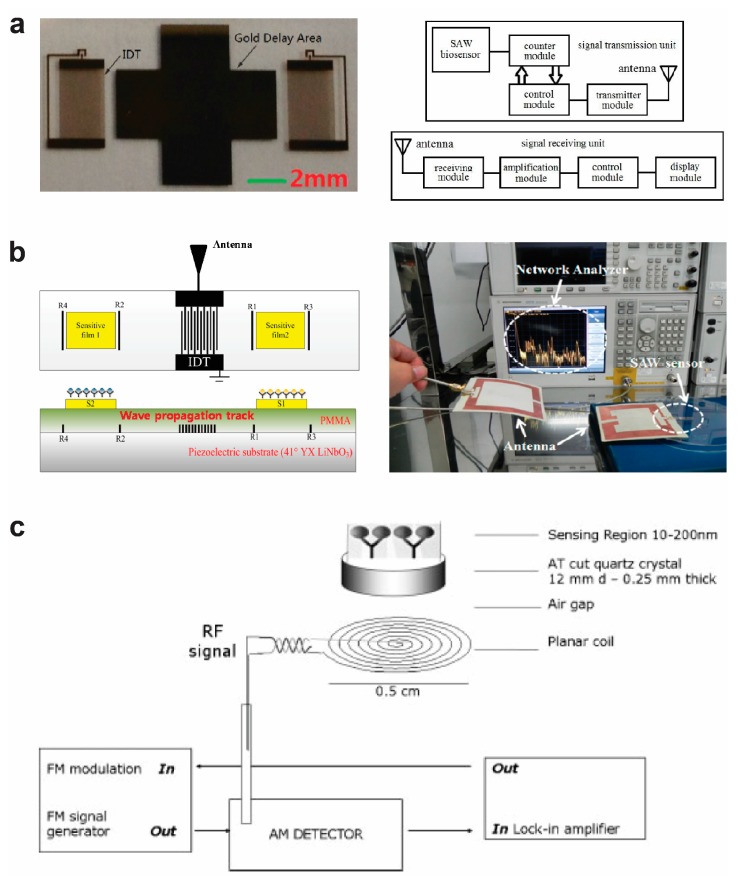
Wireless acoustic-based bioelectronic sensors. (**a**) Two-port SAW-based biosensor with antenna. Reprinted with permission from [64]. Copyright (2014) Worldscientific. (**b**) One-port SAW-based biosensor with antenna [65]. (**c**) Magnetic acoustic resonance biosensor. Reprinted with permission from [42]. Copyright (2006) Wiley.

Figure 2a shows a two-port SAW-based wireless biosensor, using a delay line to transmit the signal [64]. The gold interdigital electrodes and the center gold delay area are fabricated onto a piezoelectric substrate by microfabrication. To transmit the signal, a transmitting block with an antenna and a receiving block with an antenna are separately integrated on a printed circuit board (PCB). The signal information in the SAW is processed in the transmitting block and transferred from the antenna in the transmitting block to the antenna in the receiving block, which is further processed to be displayed in a light-emitting diode display. The mass loading on the SAW changes the frequency.

Figure 2b shows a one-port love mode SAW wireless biosensor for the detection of two analytes, using reflective delay lines to transmit the signal [65]. The inter-digital electrode and grating reflectors are fabricated onto a piezoelectric substrate. A poly(methyl methacrylate) (PMMA) layer is used as the waveguide layer. Gold electrodes are deposited onto the PMMA layer and functionalized with bioreceiptors. In the presence of target analytes, the analytes bind to the specific bioreceptors to change the mass loading on the sensor that slows down the propagating velocity of the acoustic wave and shifts the reflection peak in the time domain. Two planar antennas are used to transmit the signal, one is connected to the SAW sensor, the other is connected to the network analyzer, and the signal can be transmitted from the SAW sensor to the network analyzer wirelessly.

Figure 2c shows a magnetic acoustic resonance biosensor [42]. In the magnetic acoustic resonance sensor, a planar spiral coil is connected to a radio frequency (RF) signal generator, an amplitude-modulation (AM) detector, and a lock-in amplifier. The coil is positioned under the surface of a plate that can generate a transverse acoustic wave by an electromagnetic field and functions as the sensing transducer. In the presence of a target analyte, the analyte changes the surface of the plate that further affects the propagation path and results in changes in velocity, frequency, or amplitude of the wave.

The sensing transducers of acoustic sensors generally need to possess certain properties to enable the acoustic phenomena for the sensors, and this limits the choices of the transducer substrates. Until now, very few studies have combined the wireless antennas and the acoustic-based sensors to develop wireless bioelectronic sensors. It is expected that integration of wireless antennas with SAW sensors or magnetic acoustic resonance sensors will lead to new devices.

## 5. Wireless Magnetoelastic Biological Electronic Sensors

Applying an alternating electromagnetic field on a magnetoelastic material is another method for the construction of a wireless bioelectronic sensor.

**Figure 3:**
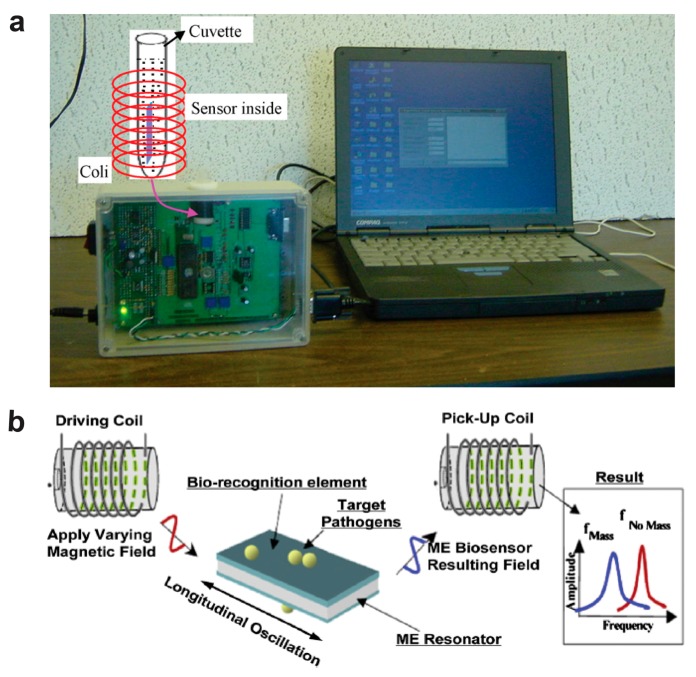
Wireless magnetoelastic bioelectronic sensors. (**a**) Experimental setup about microprocessor-based electronic box. Reprinted from [66]. Copyright (2010), with permission from Elsevier. (**b**) Operating principle of the magnetoelastic biosensor. Reprinted from [68]. Copyright (2012), with permission from Elsevier.

Figure 3a shows the experimental setup for a microprocessor-based magnetoelastic sensor [66]. A magnetoelastic ribbon is placed in a cuvette containing a buffer solution and the target analyte. The cuvette is inserted within a solenoid coil that is used for signal telemetry. A reader in the microprocessor-based box is used to measure the resonance frequency from the magnetoelastic ribbon. The sensor in the cuvette does not require a precise alignment and a physical connection to the detector, and the signal is transferred wirelessly. In addition to the intracoil detection method, extracoil detection is studied. A flat scanning coil has been developed that enables measurement of magnetoelastic biosensors placed on surfaces. The magnetoelastic biosensors are dropped onto the surface, and a handheld coil with a transmission distance of a few millimeters is manually used to excite and read the magnetoelastic sensors [43,67].

Figure 3b shows the operating principle of a magnetoelastic biosensor [68]. An exciting coil generates a magnetic field, and the magnetoelastic ribbon vibrates mechanically at a characteristic resonance frequency by converting a magnetic energy to a mechanical energy, and this generates a magnetic flux that can be detected with a pick-up coil remotely. The resonance frequency is related to the properties and dimensions of the magnetoelastic sensor, and mass loading on the sensor surface changes the resonance frequency. From a mass change, the quantity of the analyte can be determined.

Magnetoelastic sensors have been used to construct several types of biosensors. Different types of bioreceptors can be immobilized on the magnetoelastic ribbons. The bioreceptor-functionalized magnetoelastic material can generate a characteristic resonance frequency. In the presence of target analytes, the analytes bind to the bioreceptors on the magnetoelastic ribbon, which decreases the characteristic resonance frequency. By taking advantage of this change, several magnetoelastic biosensors have been developed for the detection of glucose [58], *Staphylococcal enterotoxin* B [23], acid phosphatase [69], mycoplasma genitalium growth [70], antibacterial activity of antibiotics tetracycline and levofloxacin [70], *Bacillus anthracis* spores [54], *Escherichia coli* O157:H7 [66], and *Salmonella typhimurium* [67,71,72].

Both intracoil and extracoil-based magnetoelastic sensors need the scanning and pick-up coils to be very near to the sensors, and this limits the use of these sensors for some applications that require long-distance transmissions. Magnetoelastic sensors have been developed for the wireless detection of several analytes, and it is anticipated that more sensors for the detection of other analytes can be developed in the future.

## 6. Wireless Self-Powered Biological Electronic Sensors

Self-powered wireless bioelectronic sensors that do not require external power have attracted a great amount of attentions. However, until now, there are limited studies about the self-powered bioelectronic sensors. The wireless transmission of an electrical signal needs a power, and it indicates that there is a power conversion inside the integrated sensing system. The presence of an analyte needs to induce a power conversion inside the sensing system in order to transmit the generated signal out of the system.

A biofuel cell is a clean energy source that generally utilizes enzymes and microorganisms as electrocatalysts to oxidize its fuel to generate an electrical power. To construct a self-powered sensor, the analyte is generally the fuel, and through catalyzing biochemical processes involving the analyte, a biochemical energy is converted into an electrical energy that can further power the transmitter to send out wireless signals.

**Figure 4:**
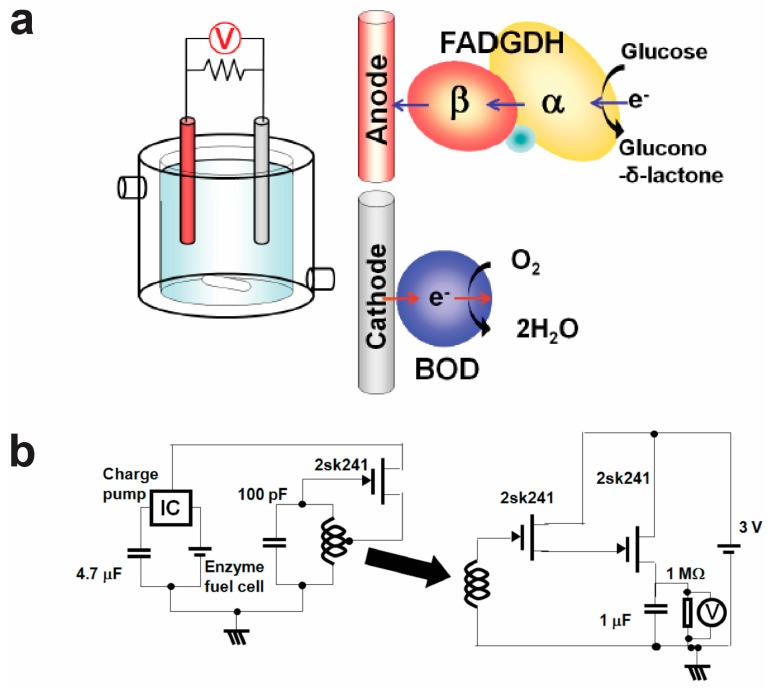
A wireless self-powered bioelectronic sensor, (**a**) a biofuel cell based on a FADGDH complex-based anode and a BOD-based cathode, and (**b**) a bioradio-transmitter circuit for the self-powered wireless sensor. Reprinted with permission from [73]. Copyright (2011) Diabetes Technology Society.

Figure 4a shows the principle of a biofuel cell based on a flavin adenine dinucleotide-dependent glucose dehydrogenase (FADGDH) complex-based anode and a bilirubin oxidase (BOD)-based cathode [73]. The anode and cathode electrodes are immersed to a cell containing a buffer solution. Glucose, as the analyte, is added into the cell, and through the biocatalytic reaction, it further generates a voltage between the anode and cathode electrodes.

Figure 4b shows a bioradio-transmitter circuit for the self-powered wireless sensor [73]. In the presence of the analyte, glucose, an electrical power is generated in the biofuel cell through the biological reaction. To transmit the power out, a capacitor, a charge-pump circuit, and an oscillator circuit are connected to the biofuel cell. The power from the biofuel cell charges a capacitor via the charge pump circuit. The time or frequency during the charge and discharge cycles of the capacitor determines the concentration of the analyte. The signal is transmitted by a mutual inductance between the two coils. The receiver receives, amplifies, and converts the signal.

The biofuel cell-based self-powered biosensor does not require any external energy input, and it provides a new device configuration for biosensor construction. However, the types of the target analytes that can be used in biofuel cells may be limited, since the majority of the analytes cannot serve as the fuels.

## 7. Wireless Potentiostat Systems for Biological Electronic Sensors

Wireless potentiostat systems are based on integrating wireless antennas with potentiostats and, recently, developing these biosensing systems has attracted great interests [2,74,75,76,77,78,79,80,81,82]. The function of a wireless-based potentiostat for powering a sensing transducer is the same as a wire-based potentiostat in a conventional biosensor. For a wireless bioelectronic sensor that is constructed by using a wireless potentiostat, the sensing transducer is generally an electrochemical sensor. The wireless antenna can transfer the data from the potentiostat to a computer, a smart phone, or a tablet wirelessly. The wireless communication approach can be based on RFID, Bluetooth, Wireless Fidelity (Wi-Fi), or Global System for Mobile Communication. RFID and Bluetooth have been mainly studied for the construction of wireless potentiostats.

**Figure 5:**
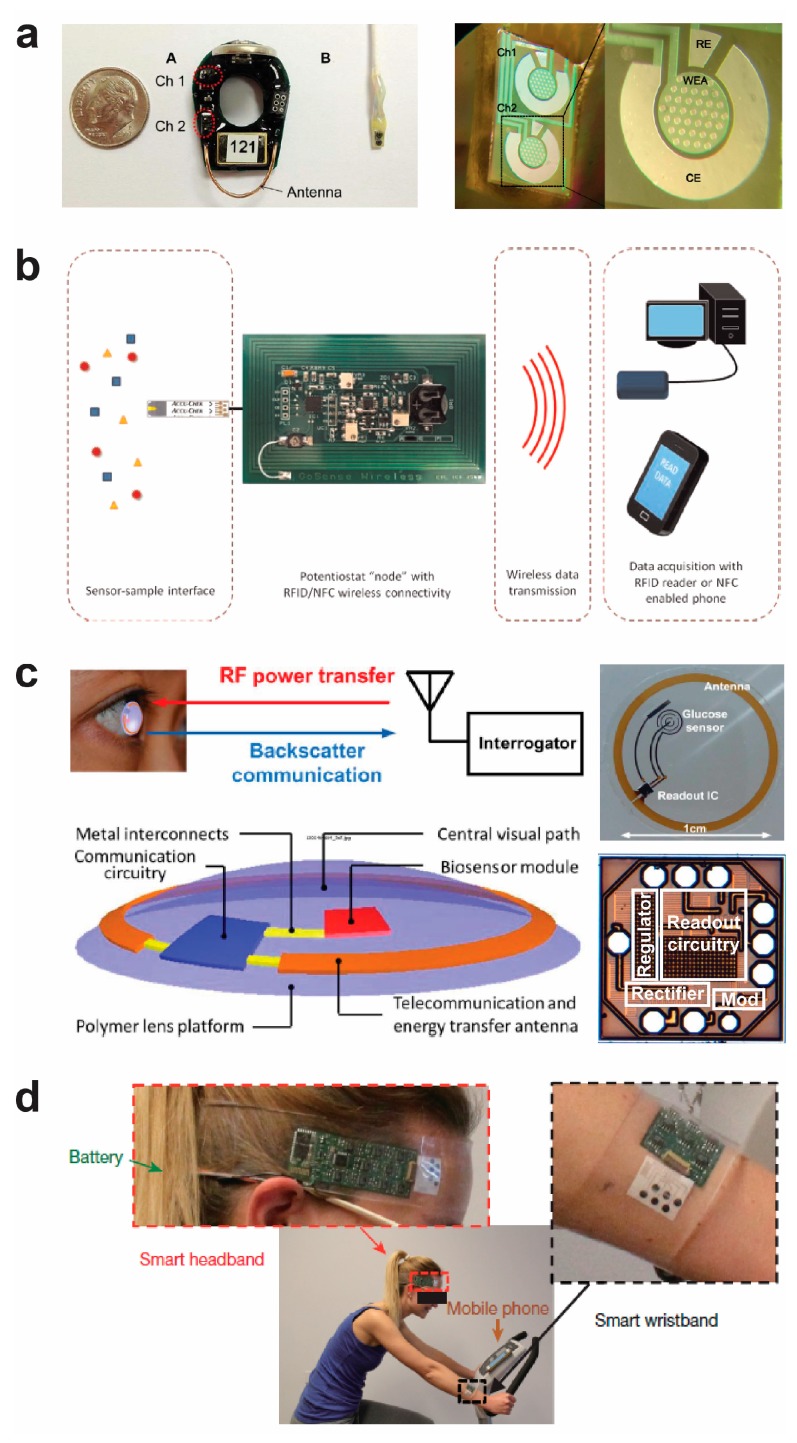
Wireless “potentiostat” system-based bioelectronic sensors. (**a**) Pinnacle 8151 wireless potentiostat with two independent electrochemical cells. Reprinted with permission from [74]. Copyright (2014) IEEE. (**b**) A wireless biosensor based on near-field communication. Reprinted from [75]. Copyright (2015), with permission from Elsevier. (**c**) A noninvasive wireless sensor platform on a contact lens for the wireless detection of glucose in tear film. Reprinted with permission from [76]. Copyright (2012) IEEE. (**d**) A wearable sensor that integrates electrochemical sensor arrays, circuits, and Bluetooth on a flexible printed circuit board for the detection of biomarkers in sweat [77]. Reprinted by permission from Macmillan Publishers Ltd: [Nature] (Gao et al. 2016), copyright (2016).

Figure 5a shows a Pinnacle 8151 wireless potentiostat with two channels (Figure 5a left), and the sensing electrodes (Figure 5a right) [74]. In the presence of analytes, the analytes can generate signal changes on the sensing electrodes, and the small wireless potentiostat can detect these signals and process the signals to further wirelessly transmit these signals on a 916 Hz band to a central base station [74].

Figure 5b shows a circuit board for the wireless communication between a biosensor to a mobile system based on the near-field communication (NFC) standard, a special type of RFID that operates at 13.56 MHz [75]. The electrochemical current signal is collected, and the analog signal is converted to a digital signal that is wirelessly transferred to a smart phone, a tablet, or a computer.

Figure 5c shows a noninvasive wireless sensor platform on a contact lens for the wireless detection of glucose in a tear film [76]. The sensor system consists of an electrochemical glucose biosensor, a low power readout integrated circuit (IC), and a loop antenna for power and data transfers. The system is wirelessly powered using RF power at 1.8 GHz sent from an interrogator at a distance of 15 cm. The IC chip processes and converts the signal to power the electrochemical sensor. In the presence of glucose, glucose can change the electrical signal of the sensor, which is further processed by the IC and read by the external reader.

The above wireless bioelectronic sensors are mainly based on the combination of electrochemical sensors, integrated circuits, and RFID. Though some of the sensors are based on flexible substrates, the integrated circuits are based on conventional hard substrates. Figure 5d shows a wearable sensor that integrates electrochemical sensor arrays, integrate circuits, and Bluetooth on a flexible printed circuit board for the detection of biomarkers in sweat [77]. A DC current signal is generated from the sensing transducer, processed by the circuits, and transmitted by the Bluetooth. Bluetooth for wireless communications enables the signal transmission from a wearable sensor to a nearby mobile phone.

Wireless potentiostat-based bioelectronic sensors may play significant roles and open up significant opportunities for the development of future wireless biosensing systems. The sensing systems confine the sensing transducers and signal processing units within sheet-based small areas. These sensing systems can be more flexible for portable or real-time monitoring of target analytes. Though, until now, only a few of these types of sensors have been developed, and it is expected that more wireless bioelectronic sensors can be developed based on the wireless potentiostats.

## 8. Conclusions and Future Perspectives

Effective interfacing of wireless antennas and bioelectronic systems has been a great challenge in the development of wireless biosensors. Recently, there have been attempts to study wireless bioelectronic systems, including RFID-based biosensors, acoustic wave-based biosensor, magnetoelastic-based biosensor, self-powered biosensors, and wireless potentiostat-based biosensors. These introduce new opportunities in wireless bioelectronic sensors. Wireless bioelectronic sensors could be used in a variety of fields, including healthcare, environmental monitoring, food quality control, and defense. According to different application, the design of the anticipated wireless bioelectronic sensor for a specific application could be different. The major catalysts for these exciting advances are (1) the design of efficient wireless antennas; (2) the exploration of effective biosensing transducers; and (3) the successful integration of biosensing transducers and wireless antennas. Additionally, there are several other factors that may affect the detection performance, such as the amplitude of an input signal into the sensing system, and an AC or DC sensing signal. The field is nascent, and to date, there are only a few methods to integrate wireless antennas and biosensing transducers to construct wireless bioelectronic sensors for the detection of a few analytes. It is anticipated that there is a large space for the integration of wireless antennas and biosensing transducers to be explored in the future to develop new wireless bioelectronic sensors for a wide range of applications.
